# Medical Charity Fund of Lower Canada

**Published:** 1845-01

**Authors:** 


*Medical Charity Fund of Lower Canada.*—Reference was recently made, in the Journal, to the late anniversary of the Medical Charitable Association of New York. A similar society has been organised in Lower Canada, to meet alternately at Quebec and Montreal. The object is to afford relief to distressed physicians, caused by inability to practice, either through age or infirmity; and, also, to relieve the widows and orphans of physicians. With a view to raising a fund to draw upon in after times, the society proposes not to begin to extend relief till the expiration of ten years from this time. Each member is to pay, yearly, into the treasury, £3, which in ten years will amount to £4,558, interest included, provided 100 members are joined. At the end of the ten years there would be at the disposal of the committee of relief £285, the interest of the capital.

This is a spirited, humane movement, honorable to the profession of Canada. It is strange that the practitioners of Massachusetts, but especially those of Boston, cannot be roused to action in this important matter. Surely there are enough in active business, who might easily appropriate a small sum annually, for a fund to feed and clothe the unfortunate of their own profession. We have urged the matter so often, that a feeling of discouragement comes over us, and especially when we witness the exertions of others, in every direction, in such a noble charity.

*Boston Med. and Surg. Jour.*

